# Ultra-high-pitch photon-counting computed tomography (PCCT) versus ultra-high-pitch dual-source computed tomography (DSCT) for coronary artery disease evaluation in transcatheter aortic valve implantation (TAVI) patients

**DOI:** 10.1016/j.ejro.2026.100824

**Published:** 2026-10-28

**Authors:** Ines Mecking, Marc von der Stück, Eric Corban, Michael Frick, Jörg Schröder, Roman Iwa, Christiane K. Kuhl, Timm Dirrichs

**Affiliations:** a Department of Diagnostic and Interventional Radiology, University Hospital RWTH Aachen, Pauwelsstraße 30, Aachen, 52074, Germany; b Department of Cardiology, Angiology and Internal Intensive Care, University Hospital RWTH Aachen, Pauwelsstraße 30, Aachen, 52074, Germany

**Keywords:** Coronary Imaging, Cardiac CT, Photon Counting Computed Tomography, TAVI

## Abstract

**Rationale and objectives:**

To compare radiation dose and image quality of coronary artery reconstructions obtained from non–coronary-dedicated transcatheter aortic valve implantation (TAVI) planning CT scans using ultra-high-pitch photon-counting CT (PCCT) and ultra-high-pitch energy-integrating detector dual-source CT (EID-DSCT; hereafter referred to as DSCT), including assessment of the feasibility of coronary artery evaluation according to the Coronary Artery Disease Reporting and Data System (CAD-RADS).

**Materials and methods:**

Retrospective study on consecutive patients with aortic valve stenosis (AS) undergoing contrast-enhanced TAVI planning CT, either PCCT or DSCT between August 2021 and June 2023. CT dose index (CTDI) and dose length product (DLP) were used to calculate the effective radiation dose (eRD). Signal-to-noise ratio (SNR) and contrast-to-noise ratio (CNR) were calculated using standardized region-of-interest (ROI) analysis. Coronary arteries were evaluated by three independent radiologists using 5-point Likert scales followed by a classification according to the CAD-RADS system.

**Results:**

187 patients (83 female, median age 82, IQR: 76–84) underwent 190 CT-examinations, 103 PCCT (54.2%) and 87 (45.8%) DSCT. Three patients underwent both PCCT and DSCT for clinical reasons. Mean CTDI and DLP were significantly lower in PCCT compared to DSCT (21.9 vs. 52.8 and 607.9 vs. 906.8, respectively; both p < 0.001), with a correspondingly lower mean effective radiation dose (eRD) in PCCT compared to DSCT (10.3 ± 5.4 mSv vs. 15.4 ± 5.6 mSv, p < 0.001). While both SNR and CNR were lower in PCCT than in DSCT (SNR: 35.28 ± 11.63 in PCCT vs. 45.6 ± 16.3 in DSCT, p < 0.001; CNR: 49.25 ± 13.31 in PCCT vs. 57.42 v ± 22.55 in DSCT, p = 0.004), PCCT outperformed DSCT in every one of the eleven visual image categories. Overall image quality was rated as good or excellent in 71.8% (74/103) of PCCT vs. 19.5% (17/87) of DSCT scans. Coronary reconstructions were rated as good or excellent in 72.8% (75/103) of PCCT vs. 20.7% of DSCT datasets. CAD-RADS [1–5] rating was possible in 68.9% (71/103) of PCCT vs. 39.1% (34/87) of DSCT datasets.

**Conclusion:**

Photon-counting computed tomography (PCCT) allows for significantly improved assessability of the coronary arteries in TAVI planning CT compared with dual-source CT (DSCT), while achieving a substantially lower radiation dose.

## 1 Introduction

Increasing global life expectancy has been accompanied by a growing burden of degenerative aortic valve stenosis (AVS), a leading cause of valvular heart disease worldwide, with the highest age-standardized prevalence in high-income regions and the most pronounced recent increases in middle- and low-to-middle sociodemographic regions [1], [2], [3], [4], [5]. Degenerative/calcific AVS shares several established cardiovascular risk factors and pathobiological features with atherosclerotic disease [5], [6]. Consequently, the number of patients requiring valve replacement continues to increase. The introduction of transcatheter aortic valve implantation (TAVI) has expanded treatment options beyond surgical aortic valve replacement (SAVR), particularly for older and higher-risk patients. Current ESC/EACTS and ACC/AHA guidelines recommend TAVI as an established alternative to surgical aortic valve replacement, particularly in older patients and those at increased surgical risk [7].

Transcatheter aortic valve implantation (TAVI) has become an established treatment option for patients with severe aortic stenosis, particularly in older patients and those at increased surgical risk [8], [9]. Accurate preprocedural assessment of concomitant coronary artery disease (CAD) is clinically relevant, as significant CAD may necessitate further invasive evaluation or revascularization, including percutaneous coronary intervention (PCI) or coronary artery bypass grafting (CABG). While invasive coronary angiography (ICA) has long served as the reference standard, computed tomography coronary angiography (CTCA) has emerged as a non-invasive alternative, with meta-analyses demonstrating diagnostic accuracy comparable to ICA in low-to-intermediate-risk patients while offering lower procedural risks and costs [10], [11], [12], [13], [14]. The CAD-RADS classification system further enables standardized reporting of coronary CT findings, facilitating risk stratification and clinical decision-making [15].

Preprocedural CT is an essential component of TAVI planning, providing comprehensive assessment of the aortic annulus, aortic root anatomy, coronary ostia, and vascular access route [8], [9], [10], [13], [16], [17]. Incorporating coronary assessment into the TAVI planning CT could therefore provide additional clinically relevant information and facilitate a comprehensive “one-stop-shop” approach [18], [19]. However, CT-based TAVI planning presents unique challenges for coronary artery assessment. Unlike dedicated CCTA, TAVI planning CT is typically performed without dedicated coronary premedication, including routine heart-rate control and vasodilation. In particular, sublingual nitrates are generally contraindicated in patients with severe aortic stenosis, while beta-blockers are not routinely administered. Moreover, the examination must provide adequate visualization of the coronary arteries while simultaneously covering the entire aortic root and arterial access route within a single contrast-enhanced acquisition, potentially limiting coronary image quality [16]. These challenges may be further pronounced in patients with extensive coronary calcification, in whom conventional energy-integrating detector (EID) CT may be affected by reduced contrast resolution, blooming and streak artifacts, stenosis over-estimation and subsequent non-diagnostic coronary segments [18].

Photon-counting CT (PCCT) may be particularly advantageous in this setting compared with conventional EID CT. PCCT has demonstrated improvements in image quality and artifact reduction compared with conventional EID-CT [20], [21], [22], [23], [24], although quantitative image-quality metrics may depend on acquisition and reconstruction parameters [18].

These properties may improve coronary assessment in TAVI CT, where coronary motion, the absence of routine premedication, and extensive coronary calcification can limit image interpretability.

Although recent studies [25], [26], [27] have explored the use of PCCT for coronary assessment during TAVI work-up, evidence regarding its impact on coronary image quality and vessel assessability within routine, non-coronary-dedicated TAVI CT remains limited, particularly in comparison with contemporary dual-source CT and with respect to standardized CAD-RADS classification.

Therefore, this study aimed to compare coronary image quality and assessability in non-coronary-dedicated ultra-high-pitch TAVI CT performed using PCCT and dual-source CT (DSCT), including the feasibility of standardized CAD-RADS classification.

## 2 Materials and methods

Retrospective study under IRB-approval of the authors` institution (EK 205–17) and in accordance with the Declaration of Helsinki. Patients were informed in detail about CT examinations and the respective risks and limitations, and written informed consent was provided afterwards.

### 2.1 Participants

A total of 200 consecutive patients undergoing CT for TAVI planning between August 2021 and June 2023 were included in this single-center study. Patients were referred by the in-house departments of Cardiology or Cardiac Surgery. The decision to perform CT was jointly agreed on a multi-disciplinary board consisting of cardiologists, cardiac surgeons and board-certified radiologists.

### 2.2 Inclusion and exclusion criteria

Patients were eligible for inclusion if they were adults (>18 years) with clinically and sonographically confirmed aortic valve stenosis who had undergone preprocedural CT for TAVI planning. Patients with a history of coronary artery bypass grafting (CABG) were excluded from coronary artery analyses because of the anticipated high coronary plaque burden and presumed reduced assessability of the native coronary arteries.

Patients with a history of percutaneous coronary intervention (PCI) and coronary stent implantation were considered eligible for visual coronary assessment and CAD-RADS classification but were excluded from coronary artery calcium assessment.

### 2.3 Imaging protocol

Patients underwent contrast-enhanced aorto-iliac CT for clinically indicated TAVI planning, using either photon-counting CT (PCCT) or dual-source CT (DSCT). Three patients underwent both PCCT and DSCT due to clinical indications. Between August 2021 and September 2022, CT examinations were performed on a third-generation dual-source CT (SOMATOM Force, Siemens Healthineers). Between September 2022 and June 2023, CT examinations were performed on a first-generation PCCT system (NAEOTOM Alpha, Siemens Healthineers).

As previously published [28] and in accordance with the Recommendations for IV Contrast Administration of the Society of Cardiovascular Computed Tomography Expert Consensus Paper [29], contrast media with an iodine concentration of 370 mg iodine/mL (iopromide, Ultravist 370, Bayer Vital) was administered through an 18-gauge cubital vein catheter with a flow rate of 5 mL/s and a volume of 1 mL per kilogram of body weight. Bolus tracking was applied to determine the time of peak enhancement in the ascending aorta. Each contrast bolus was flushed with the same amount of physiological saline solution (0.9% NaCl). All scans were acquired during inspiratory breath-hold with patients positioned supine and arms elevated above the head.

The CT acquisition protocol utilized for both scanners consisted of a prospectively ECG-triggered high-pitch CT scan ranging from the thorax to the femoral vessels.

Contrast enhanced PCCT images were acquired as dual source, multi-energy, high-pitch helical scans with a pitch factor of 3.2 and a collimation of 2 x 144 × 0.4 mm. Peak tube voltage was set at 120 kVp. CARE k(e)V automatic exposure modulation was set to “full” and setting "vascular". Image Quality (IQ) Level was set at 100. Phase start was set at 35% of the RR-interval [20].

Contrast enhanced DSCT images were acquired as dual-source, single-energy, high-pitch helical scans with a pitch factor was of 3.2 and a collimation of 2 × 192 × 0.6 mm. Z-flying focal spot oversampling was enabled. The effective detector collimation was identical to that of the utilized PCCT (DSCT=96 × 0.6 mm, PCCT=144 × 0.4 mm). The quality reference point was set to 320 mAs/rot at 120 kV with CARE kV set to “vascular” mode. Phase start was set at 35% of the RR-interval, as previously described [20]. Detailed image acquisition and CT system specifications are provided in Table 2.

### 2.4 Image reconstruction

Images were reconstructed with section thicknesses of 5.0, 1.0, and 0.6 mm and reviewed in axial, sagittal and coronal planes using both soft-tissue and lung window settings. For PCCT data, soft-tissue convolution algorithms were applied with a Bv44 kernel in combination with level-3 quantum iterative reconstruction (QIR). DSCT datasets were processed using a Bf32 kernel with strength 3 advanced model-based iterative reconstruction (ADMIRE). PCCT datasets were reconstructed as monoenergetic images at a fixed energy level of 65 keV, selected to provide a balanced trade-off between iodine contrast enhancement, image noise, and overall image quality, whereas DSCT images were generated using a conventional non-spectral reconstruction technique, yielding contrast characteristics representative of the underlying materials (e.g., iodine, calcium, and soft tissue) and dependent on the applied tube voltage.

Maximum intensity projections (MIPs) were created in sagittal and coronal planes. Three-dimensional reconstructions of the heart, thoracic and abdominal aorta, and iliac arteries, as well as curved MIP visualizations of the transfemoral access route, were performed using dedicated post-processing software (Syngo.Via, VB60A HF03; Siemens Healthineers). Runoff analyses and curved planar reconstructions were performed with the same software.

### 2.5 Quantitative assessment of Image SNR and CNR

Signal-to-noise-ratio (SNR) and Contrast-to-noise-ratio (CNR) were calculated by regions of interest (ROI) with a standardized area of 1.0 cm² placed in the descending aorta and the subcutaneous adipose tissue to assess signal and noise in Hounsfield units (HU). All measurements were performed on axial image sections with a slice thickness of 5.0 mm. The SNR was determined by dividing the mean signal intensity by the standard deviation of the noise. The CNR was calculated by subtracting the mean HU of the subcutaneous fat from that of the aorta to obtain the contrast, which was then divided by the noise measured in the unenhanced images.

### 2.6 Quantitative assessment of coronary artery calcifications (CAC)

Coronary artery calcium (CAC) scoring was performed on unenhanced CT images with a slice thickness of 5 mm using dedicated software (Syngo.Via, version VB60A_HF03; Siemens Healthineers). The absolute calcium mass score (MS), expressed in milligrams (mg), was calculated by multiplying the lesion volume by the mean attenuation above a threshold of 130 Hounsfield units (HU) and calibrated using a calibration phantom containing known densities of calcium hydroxyapatite. The calcium volume score (CVS) was determined in cubic millimeters (mm³) based on an attenuation threshold > 130 HU and a minimum lesion size of 0.5 mm³ , utilizing isotropic interpolation [30]. Finally, the traditional Agatston score was calculated by multiplying the area of each calcified lesion by a density factor derived from the peak attenuation, as previously described [31].

Patients with a history of percutaneous coronary intervention (PCI) and stent implantation were excluded from calcium scoring.

### 2.7 Visual assessment of coronary arteries

Visual assessment of anonymized datasets was performed independently by three radiologists with fourteen (TD), five (MH) and five years (EC) in cardiovascular imaging. The raters were blinded to the patients’ demographic data, medical histories as well as to the CT scanner used for image acquisition. Images derived from DSCT and PCCT were presented in a randomized order. In cases of disagreement, consensus was sought.

Visual rating of individual coronary segments was performed using a 5-point Likert scale [1], [2], [3], [4], [5]. This was followed by computer-assisted stenosis evaluation using dedicated software (SyngoVia, Siemens Healthineers) and a final classification according to the CAD-RADS system. Datasets in which one or more coronary segments could not be conclusively assessed were classified as CAD-RADS N (non-diagnostic) due to limited diagnostic utility.

### 2.8 Visual assessment of overall image quality (VIQ) and diagnostic utility of coronary reconstructions

The following categories were evaluated: (A) overall assessability of the coronary ostia, (B) assessability of the right coronary artery (RCA) ostium, (C) assessability of the left coronary artery (LCA) ostium, (D) overall diagnostic utility of coronary artery reconstructions, (E) diagnostic utility of reconstructions of the [1] left anterior descending artery (LAD), [2] right coronary artery (RCA), and [3] left circumflex artery (LCX), (F) absence of artifacts, (G) vessel contrast, and (H) overall image quality. Each category was rated on a five-point Likert scale (1 = poor, 2 = limited, 3 = moderate, 4 = good, and 5 = excellent). Scores of 1–2 were considered non-diagnostic, whereas scores ≥ 3 were considered diagnostic.

Artifact burden (F) was assessed using the following criteria: 1 = artifacts rendering images non-diagnostic, 2 = substantial artifacts impairing image interpretation, 3 = moderate artifacts, 4 = minimal artifacts, and 5 = no artifacts.

### 2.9 CAD-RADS assessment

Coronary artery disease was classified according to the Coronary Artery Disease–Reporting and Data System (CAD-RADS 2.0), based on the degree of coronary artery stenosis assessed by CCTA. Stenosis severity was graded as CAD-RADS 0 (no plaque or stenosis), CAD-RADS 1 (minimal stenosis, 1–24%), CAD-RADS 2 (mild stenosis, 25–49%), CAD-RADS 3 (moderate stenosis, 50–69%), CAD-RADS 4 A (severe stenosis, 70–99% in one or two vessels), CAD-RADS 4B (left main stenosis ≥50% or three-vessel obstructive disease with stenosis ≥70%), or CAD-RADS 5 (total coronary artery occlusion). The final patient-level CAD-RADS category was determined by the most severe coronary artery stenosis identified. Non-diagnostic segments were documented using the appropriate N modifier [15].

Furthermore, the readers were asked to determine whether a CAD-RADS score could be assigned and, in cases of agreement, to provide such a score.

### 2.10 Radiation exposure

Radiation exposure was quantified using the volume CT dose index (CTDI) and the dose length product (DLP). The effective radiation dose (eRD) was derived from the formula eRD (mSv) = DLP × k, as recommended by the European Working Group for Guidelines on Quality Criteria in CT [32] with *k* representing the age-related organ weighting factor. A conversion factor of k = 0.017 was applied, corresponding to the mean of region-specific coefficients for chest (0.017 mSv/mGy·cm), abdomen (0.015 mSv/mGy·cm), and pelvis (0.019 mSv/mGy·cm). This approach follows the beforementioned publication by the European Commission [22], as well as recent comparative studies [33] and reviews [34].

### 2.11 Statistical analysis

Categorical data are given as percentages and numbers and median with IQR or mean ± SD are utilized to express continuous variables. Inter-reader agreement was assessed using Fleiss´ Kappa. The resulting Kappa was interpreted as established by Landis and Koch [25]. Values of 0.01–0.20 were equated with no or slight, 0.21–0.40 with fair, 0.41– 0.60 with moderate, 0.61–0.80 with substantial, and 0.81–1.00 with almost perfect agreement. Continuous variables were compared through their respective means between the two study groups utilizing a two-sided t-

performed statistical analysis using SPSS Statistics for Windows, Version 28.0 (IBM Corp).

## 3 Results

### 3.1 Patients´ characteristics

A total of 200 patients underwent standardized CT for TAVI planning, of whom 13 were excluded due to a history of coronary artery bypass grafting (CABG). The final cohort comprised 187 patients (83 female and 104 male; median age, 82 years [IQR, 76–84]) who underwent 190 contrast-enhanced aorto-ilio-femoral CT examinations. Three patients underwent both photon-counting CT (PCCT) and dual-source CT (DSCT) for clinical indications, with intervals of 16, 13, and 5 months between examinations. Overall, 103 PCCT and 87 DSCT examinations were performed. Table 1 shows participants´ characteristics. Fig. 1 shows a flow chart of patient enrollment and important results in PCCT and DSCT groups.

**Table 1:** Patient demographics (n = 187).

	**PCCT**	**DSCT**	***P*****value**
**Number of data sets (n)(three each of the same patients; comparison)**	103	87	
**SexMale (%)Female (%)**	63 ± 61.2 40 ± 38.8	43 ± 49.4 44 ± 50.6	
**Age (years) (*****mean*****± SD) [IQR]**	79.34 ± 6.6 [Median: 80,5; IQR: 74.8–84]	80.94 ± 7.2 [Median: 83; IQR: 78.4–85]	.11
**Body measurement (*****mean*****± SD)**			
**Weight (kg)**	77 ± 15.1 [Median: 77,5; IQR; 65–90]	77 ± 15.5 [Median: 75; IQR: 66.3–84.8]	.97
**Height (m)**	1.69 ± 0.09 [Median: 1,7; IQR: 1.6–1.8]	1.68 ± 0.09 [Median: 1.7; IQR: 1.6–1.8]	.40
**Body surface area (kg/m**^**2**^**)**	26.8 ± 4.7 [Median: 27; IQR: 23.9–29.5]	27.2 ± 4.9 [ Median: 26.4; IQR: 23.5–30.8]	.61
**Contrast Agent (mL**) **(mean ± SD)**	77 ± 15.1 [Median: 77.5; IQR; 65–90]	77 ± 15.5 [Median: 75; IQR: 66.3–84.8]	.97

All data are reported as mean, ± SD, and in part with median and IQR in square brackets.

**Table 2:** CT-Scanner and Image acquisition specifications.

	**PCCT**	**DSCT**
**Mode**	Dual-source, multi-energy	Dual-source, single-energy
**No. of sections**	2 × 144	2 × 192
**z-coverage (mm)**	144 × 0.4	192 × 0.6
**Spatial resolution (mm)**	0.11	0.24
**Temporal resolution (msec)**	66	66
**Rotation time (sec)**	0.25	0.25
**Maximum scan speed (mm/sec)**	737	737
**Pitch Factor**	3.2	3.2
**Generator power (kW)**	2 × 120	2 × 120
**Peak tube voltage (kVp)**	140	140
**Reconstruction kernel**	Bv44	Bf32
**Image Quality (IQ) Level**	100	100
**Phase start (% R-R interval)**	35%	35%

PCCT: Photon-counting computed tomography; DSCT = EID-DSCT: Energy Integrating Detector Dual-source computed tomography

**Fig. 1:**
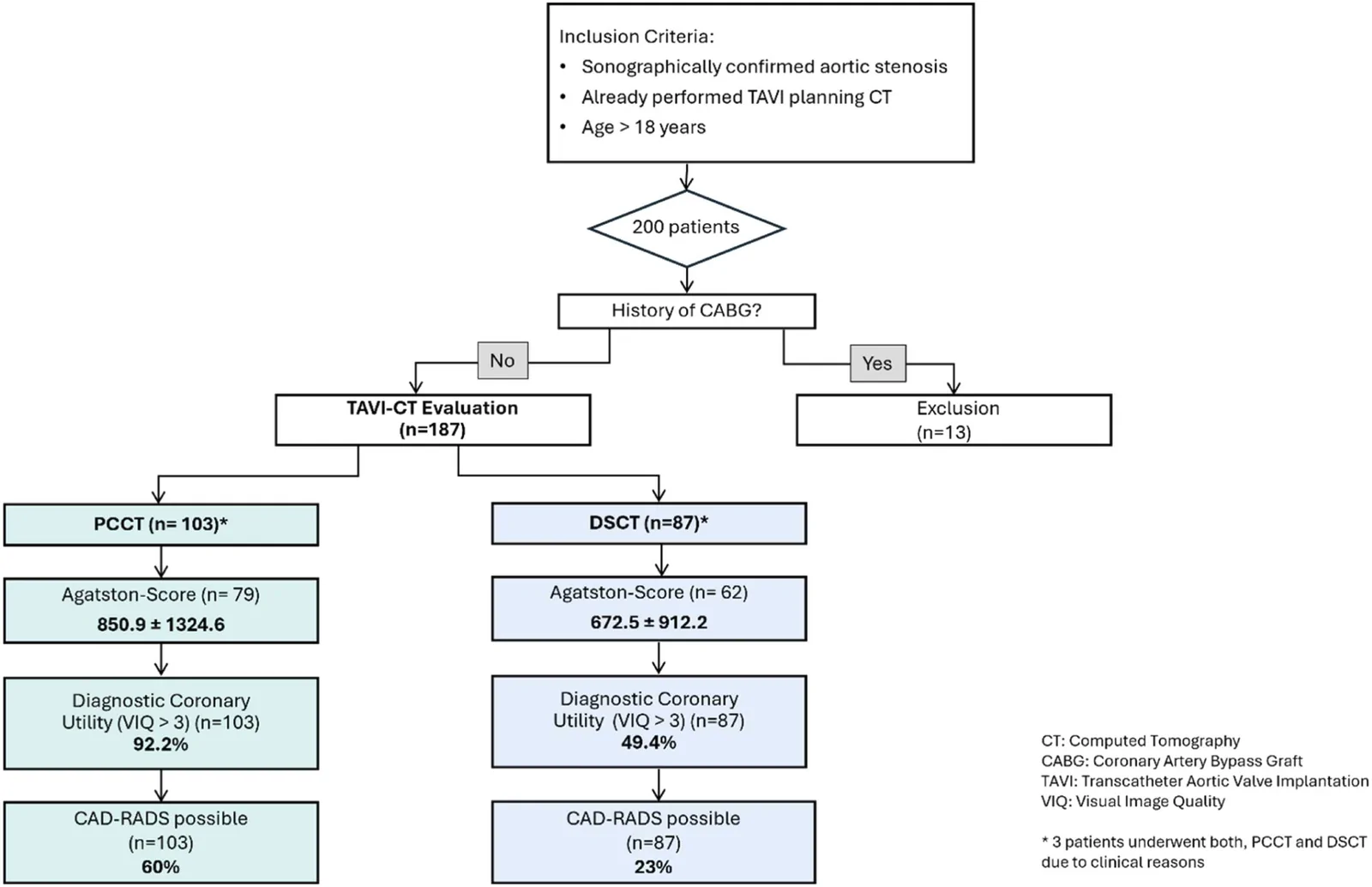
Patient Enrollment and Important Results in PCCT and DSCT groups.

### 3.2 Quantitative assessment of image SNR and CNR

SNR was significantly lower in PCCT vs. DSCT, amounting to 35.3 ± 11.6 in PCCT vs. 45.5 ± 16.4 in DSCT, p < 0.001). CNR was also lower, amounting to 49.3 ± 13.3 in PCCT vs. 57.1 ± 22.4 in DSCT, p = 0.004.

### 3.3 Quantitative assessment of coronary artery calcifications (CAC)

Quantitative Calcium Scoring was possible in 138/187 (73.8%) of patients, due to history of coronary stents in the remaining individuals. Mean CAC Agatston scores did not differ significantly between the PCCT and DSCT groups (850.9 ± 1324.6 vs. 672.5 ± 912.2, respectively; p = 0.78). Mean VCS und MS also showed no significant differences between the PCCT and DSCT cohorts, with a mean volume score of 989.3 ± 826.7 mm³ vs. 582.1 ± 995.8 mm³ (p = 0.36) and a mean mass score of 207.5 ± 196.5 mg vs. 221.7 ± 286.9 mg (p = 0.91), respectively.

### 3.4 Visual assessment of coronary arteries

General diagnostic utility of coronary reconstructions was on average rated ≥ 3 in 92.2% (95/103) of PCCT and 49.4% (43/87) of DSCT. Mean rating was 3.9 ± 0.9 (PCCT) vs. 2.8 ± 1 (DSCT) (p < 0.001). Inter-reader agreement was substantial (Kappa=0.645, IRR for each category of the scale: 1 =0.887, 2 =0.711, 3 = 0.629, 4 =0.544, 5 =0.647). PCCT also outperformed DSCT in the evaluation of each individual coronary artery (average rating ≥ 3 for diagnostic utility of LAD: 93.2% (96/103) of PCCT vs. 59.8% (52/87) of DSCT. Mean rating: 4.2 ± 0.9 (PCCT) vs. 2.9 ± 1 (DSCT) (p < 0.001); average rating ≥ 3 for diagnostic utility of RCA: 77.7% (80/103) of PCCT vs. 40.2% (35/87) of DSCT. Mean rating: 3.7 ± 1.1 (PCCT) vs. 2.7 ± 1.2 (DSCT) (p < 0.001); average rating ≥ 3 for diagnostic utility of RCX: 85.4% (88/103) of PCCT vs. 41.1% (36/87) of DSCT. Mean rating: 3.9 ± 1 (PCCT) vs. 2.8 ± 1.1 (DSCT) (p < 0.001)). On average, the general assessability of the coronary ostia/outlets was rated ≥ 3 in 94.2% (97/103) of PCCT vs. 77% (67/87) of DSCT (Mean rating: 4.5 ± 0.9 (PCCT) vs. 3.4 ± 1.1 (DSCT) (p < 0.001). Fig. 4A-4D gives an overview. Fig. 2 provides an intra-individual comparison between DSCT and PCCT of curved coronary MPRs. Fig. 3 provides an inter-individual comparison between DSCT and PCCT of curved coronary MPRs. Figs. 4A–4D provide an overview of the image quality ratings.

**Fig. 2:**
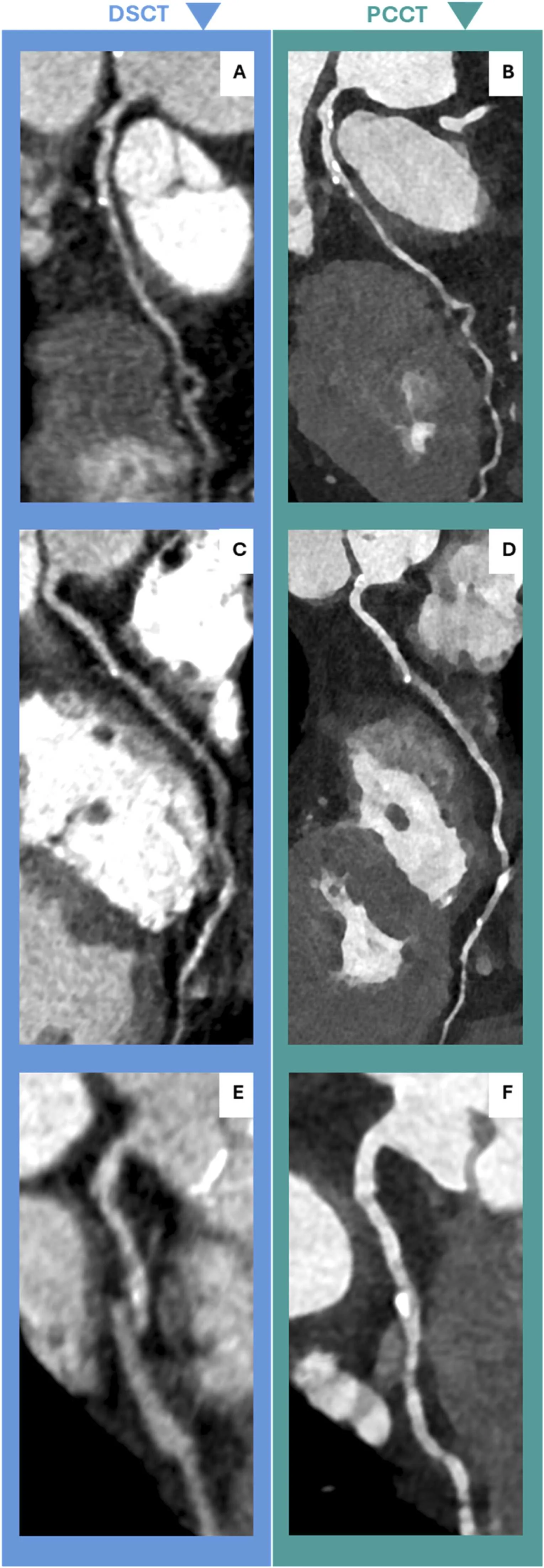
A-F: *Intra*-individual Comparison of Image Quality of Coronary Artery Curved MPRs.

**Fig. 3:**
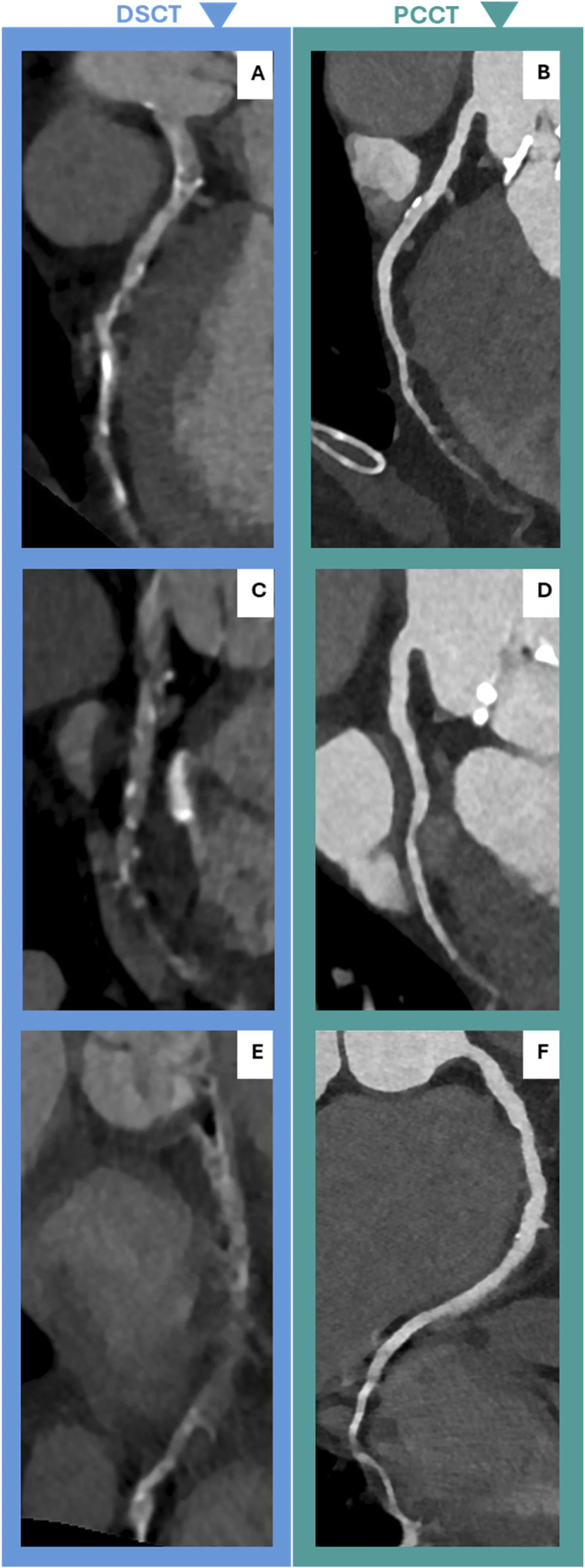
A-F: *Inter-*individual comparison of image quality of Coronary Artery curved MPRs.

**Fig. 4:**
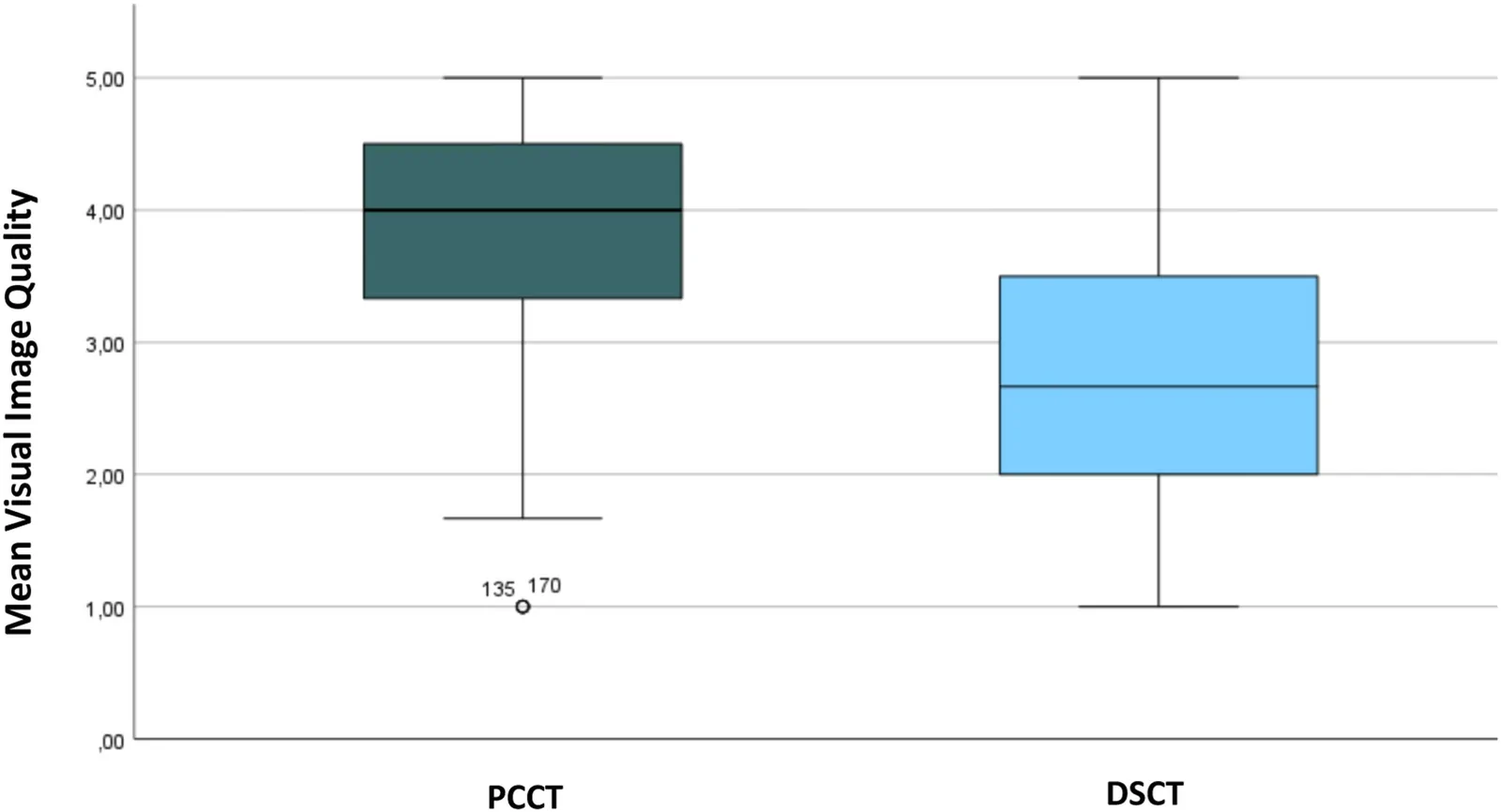

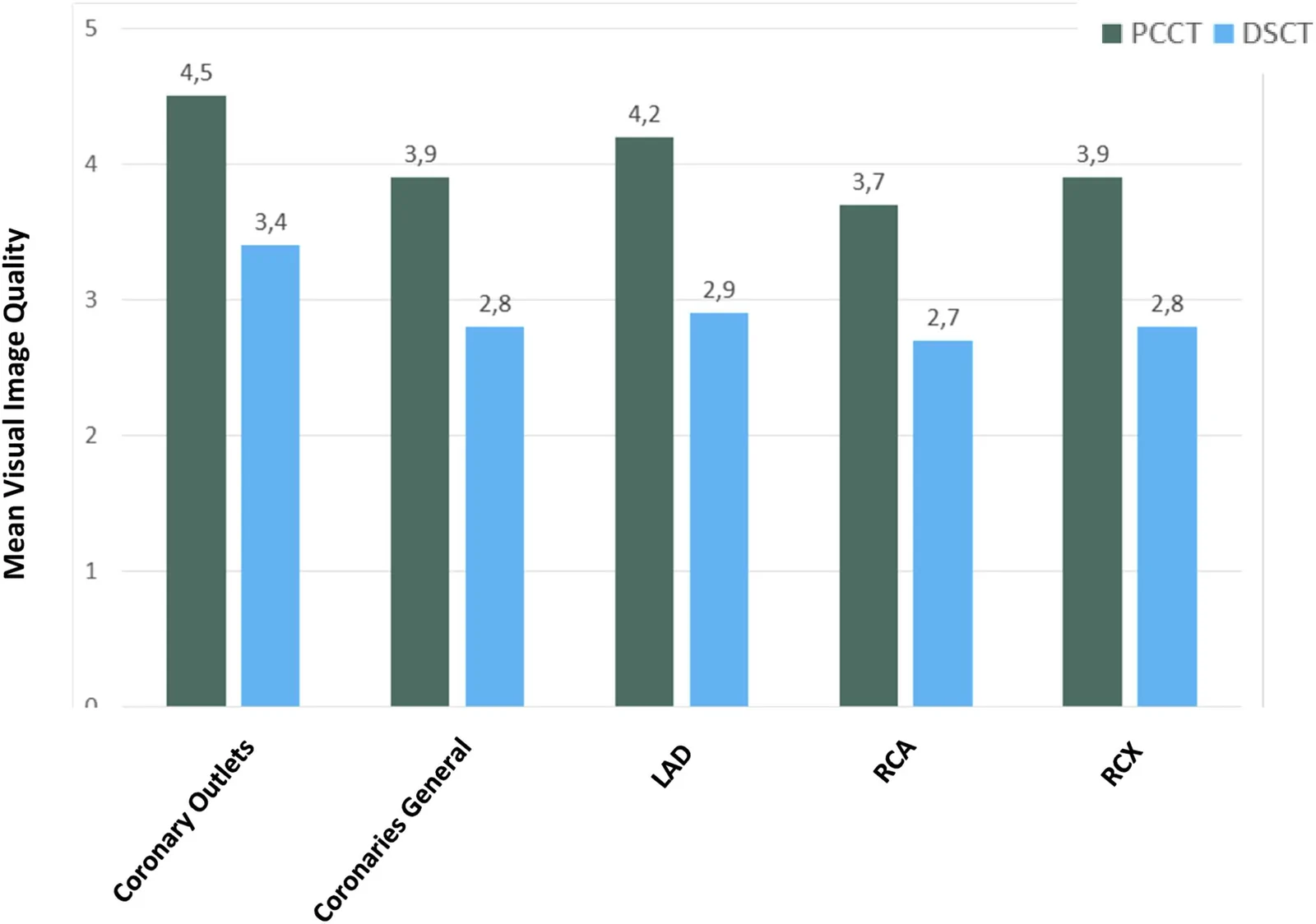

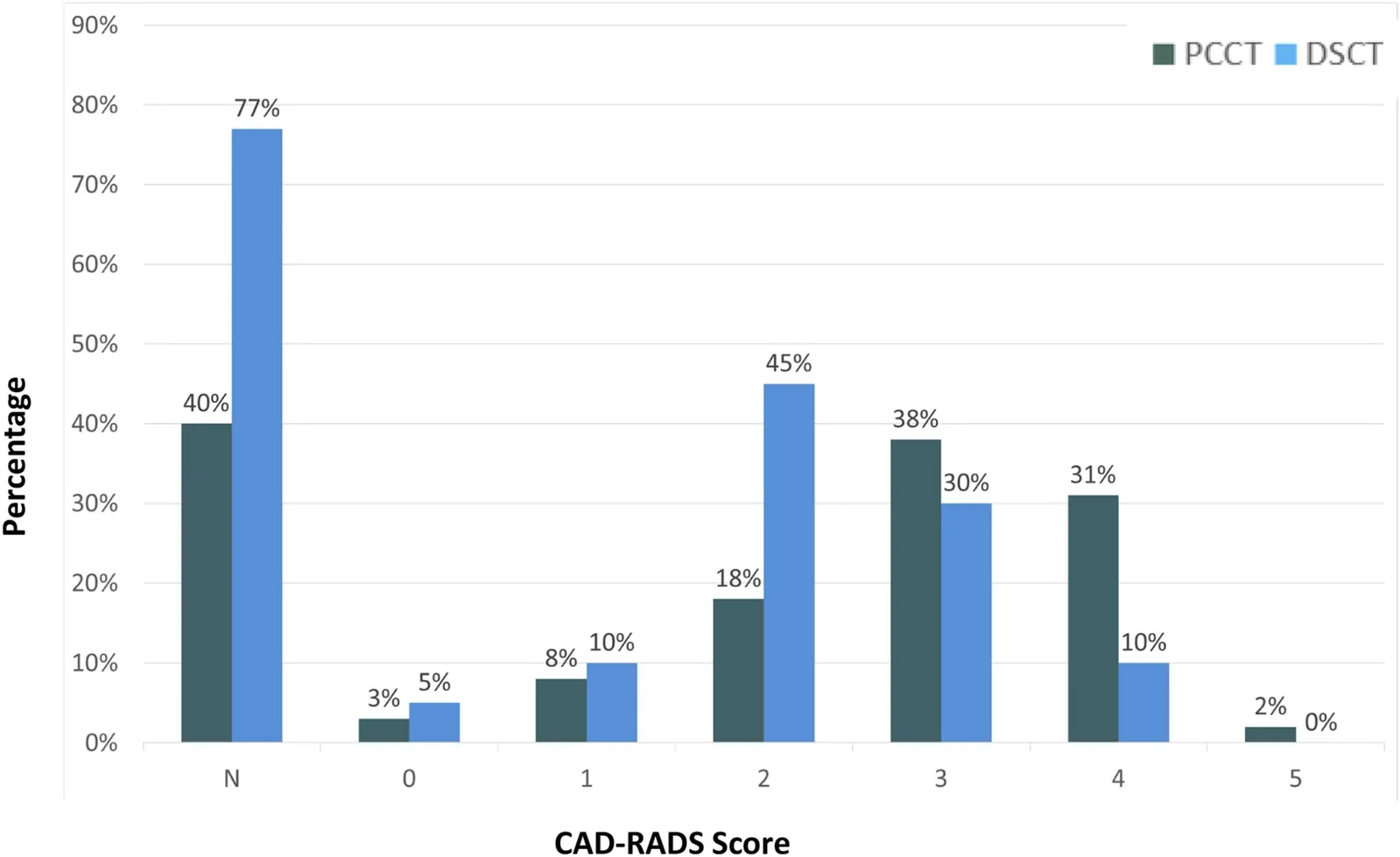

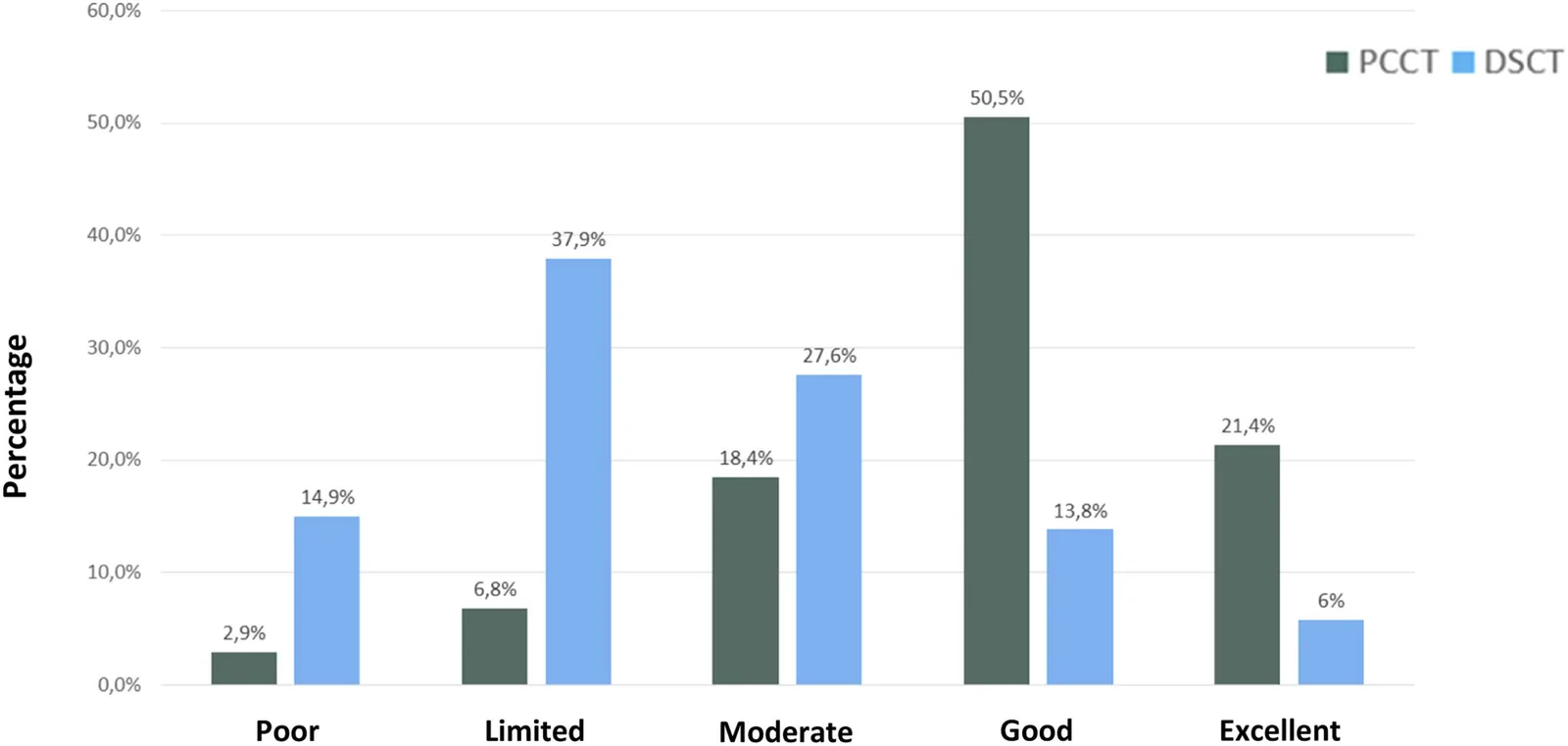
A: Visual Rating of Overall Visual Image Quality (VIQ)B: Diagnostic Utility of Coronary Reconstructions C: Distribution of CAD-RADS Scores D: Distribution of Visual Image Quality Ratings (VIQ).

### 3.5 Visual assessment of overall image quality (VIQ) and diagnostic utility of coronary reconstructions

Although quantitative SNR and CNR were lower with PCCT than with DSCT, the proportion of PCCT examinations achieving an average rating of ≥ 3 (“moderate” quality) exceeded that of DSCT in all evaluated categories (Fig. 4B). This proportion was defined as the ratio of examinations with an average Likert score of three or higher to the total number of examinations in the respective study group. Overall image quality was rated as greater than or equal to 3 (“moderate”) in 109/190 of all CT examinations. Of these 92 were executed in PCCT (89.3% of total number of PCCT examinations) and 38 in DSCT (43.7% of total number of DSCT examinations). Interrater agreement was substantial (Kappa=0.624, IRR for each category of the scale: 1 =0.905, 2 =0.798, 3 =0.562, 4 =0.485, 5 =0.562). The corresponding mean rating was 3.9 ± 0.9 (PCCT) vs. 2.6 ± 1 (DSCT) (p < 0.001).

Regarding each rating category separately, overall image quality of PCCT vs. DSCT was rated as “excellent” in 21.4% vs. 6%, “good” in 50.5% vs. 13.8%, “moderate” in 18.4% vs. 27.6%, “limited” in 6.8% vs. 37.9%, and “poor” 2.9% vs. 14.9% (See Fig. 4D)

Artifacts were less pronounced in PCCT (Mean rating: 2.3 ± 0.9 (PCCT) vs. 3.3 ± 1 (DSCT)

(p < 0.001), while vessel contrast was deemed superior (4 ± 1 (PCCT) vs. 3 ± 1.1 (DSCT) (p < 0.001).

### 3.6 CAD-RADS assessment

A CAD-RADS classification could be assigned by consensus among all three readers in 59.2% (61/103) of PCCT datasets, compared with only 23.0% (20/87) of DSCT datasets.

The distribution of CAD-RADS categories differed between the PCCT and DSCT groups (see Fig. 4C). CAD-RADS 2 was less frequent in the PCCT than in the DSCT group (18% vs. 45%), whereas CAD-RADS 3 and 4 were more prevalent in the PCCT group (38% vs. 30% and 31% vs. 10%, respectively). CAD-RADS 0 and 1 occurred at similar frequencies in both groups (3% vs. 5% and 8% vs. 10%, respectively). CAD-RADS 5 was observed only in the PCCT group (2%). The proportion of examinations classified as non-diagnostic (CAD-RADS N) was lower in the PCCT group than in the DSCT group (40% vs. 77%).

### 3.7 Radiation exposure

Mean CTDI and CLP were significantly lower in PCCT examinations than in DSCT examinations (21.9 ± 19.4 vs 52.6 ± 22.6, and 608 ± 316.7 vs. 913.4 ± 331.9, p < 0.001, respectively). Accordingly, mean effective radiation dose was also lower in the PCCT vs. DSCT group (10.3 ± 5.4 vs. 15.4 ± 5.6, p < 0.001). Distribution of DLP is depicted in Fig. 5.

**Fig. 5:**
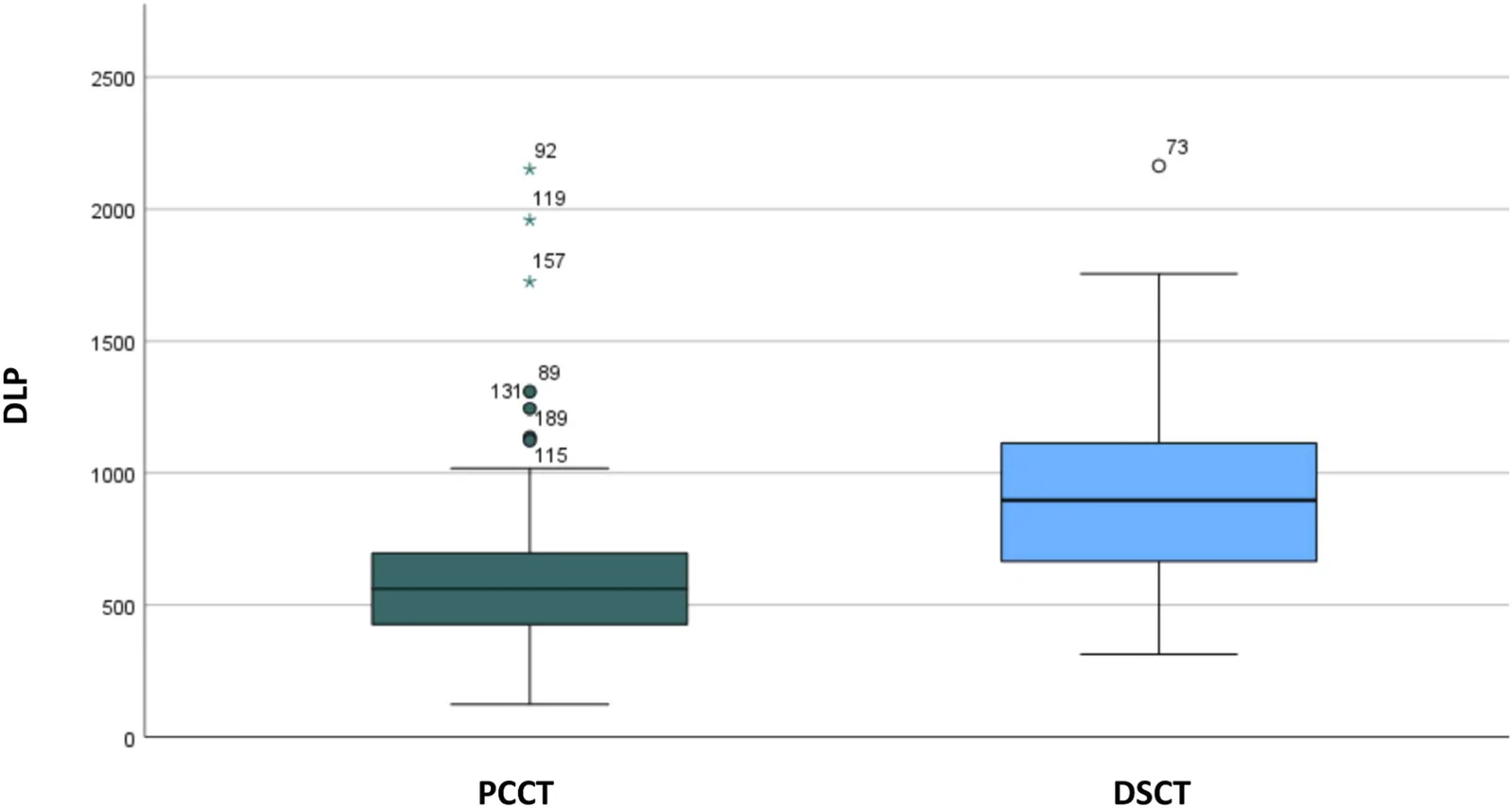
Dose Length Product.

## 4 Discussion

The present study evaluated the feasibility of coronary artery assessment in non-coronary-dedicated TAVI planning CT using photon-counting CT (PCCT) compared with dual-source CT (DSCT). Despite the absence of dedicated coronary premedication and the challenging clinical setting of patients with severe aortic stenosis, PCCT demonstrated substantially higher coronary assessability and superior subjective image quality than DSCT. This was reflected by higher visual ratings for coronary diagnostic utility, vessel contrast, and overall image quality, as well as a higher proportion of examinations in which a CAD-RADS classification could be assigned. These findings suggest that PCCT may facilitate the integration of coronary assessment into routine pre-TAVI CT examinations.

The most clinically relevant finding was the higher proportion of diagnostically assessable examinations with PCCT. A CAD-RADS classification could be assigned by all three readers in 59.2% of PCCT examinations compared with 23.0% of DSCT examinations. Of the assessable PCCT examinations, 67.2% were classified as CAD-RADS 1–3, whereas 90% of assessable DSCT examinations were classified as CAD-RADS 0–3. However, only 20.7% of all DSCT examinations yielded a classification within CAD-RADS 0–3, compared with 59.2% of all PCCT examinations. Thus, the potential clinical advantage of PCCT primarily reflects its higher rate of diagnostic assessability rather than a more favorable CAD-RADS distribution among already assessable examinations. These findings suggest that PCCT may provide useful information for clinical decision-making in a larger proportion of patients. Nevertheless, as invasive coronary angiography (ICA) was not systematically performed as a reference standard, the present study cannot establish diagnostic accuracy or demonstrate that ICA can safely be omitted.

The potential clinical relevance of improved coronary assessability is considerable in the context of TAVI planning. Coronary artery disease (CAD) is common in patients with aortic stenosis, and routine ICA adds procedural burden and carries risks, including bleeding and vascular complications [35], [36], [37], [38]. Previous studies have explored CT-based strategies to reduce unnecessary invasive testing. A multicenter study reported that CT coronary angiography (CTCA) obviated further investigations in approximately three-quarters of patients presenting with recent-onset chest pain, although substantial downstream use of ICA persisted [39]. The DISCHARGE trial found no significant difference in major cardiovascular outcomes between initial CT angiography and ICA in patients with stable chest pain and an intermediate pre-test probability of obstructive CAD [40]. Similarly, the PLATFORM study demonstrated comparable clinical outcomes and quality of life, with lower costs, for a CT angiography-based strategy incorporating selective CT-derived fractional flow reserve (CT-FFR) compared with usual care [41]. Although these studies did not specifically investigate patients undergoing TAVI, they support the broader rationale for optimizing CT-based coronary assessment. Their findings cannot, however, be directly extrapolated to the TAVI population.

Previous studies have demonstrated the potential of PCCT to improve coronary imaging, particularly through increased spatial resolution and improved characterization of coronary calcification [42], [43], [44]. In the present study, superior subjective coronary assessability was observed despite lower quantitative signal-to-noise ratio (SNR) and contrast-to-noise ratio (CNR) with PCCT than with DSCT. This apparent discrepancy highlights the limitations of conventional image-quality metrics when evaluating complex coronary structures. Visual assessability depends not only on image noise and contrast but also on spatial resolution, vessel delineation, calcium-related blooming, and other artifacts. The observed differences may therefore reflect the combined effects of detector technology and the associated acquisition and reconstruction characteristics.

Several studies have investigated coronary stenosis assessment using ultra-high-resolution (UHR) PCCT. Koons et al. and Halfmann et al. reported advantages of UHR PCCT for stenosis quantification and CAD-RADS classification [45], [46], whereas McCollough et al. found no substantial objective differences in stenosis grading compared with energy-integrating detector CT (EID-CT), despite subjective differences [47]. Wolf et al. reported a small but significant reduction in measured diameter stenosis when comparing reconstructions with similar spatial resolutions [48]. Studies comparing PCCT with ICA have further demonstrated the potential of UHR PCCT for coronary plaque characterization, calcium delineation, and stenosis quantification [49], [50], [51], [52]. In a large cohort of 7833 patients, Sakai et al. also reported fewer referrals for ICA after PCCT than after EID-CT, while revascularization was more frequent among patients who subsequently underwent ICA [42]. Collectively, these findings support the potential clinical relevance of PCCT, although differences in study populations and imaging protocols limit direct comparisons with the present study.

Our findings extend this literature to non-coronary-dedicated TAVI planning CT, in which coronary assessment is performed under less favorable conditions than dedicated coronary CT angiography. Beta-blockade is not routinely administered, and sublingual nitrates are generally avoided in patients with severe aortic stenosis because of potential haemodynamic compromise. Coronary motion, suboptimal vascular enhancement, and extensive calcification may consequently limit diagnostic assessability. The improved visual assessability observed with PCCT suggests that its image-quality characteristics may partly compensate for these challenges. Furthermore, mean Agatston, calcium volume, and calcium mass scores did not differ significantly between the PCCT and DSCT cohorts, and the PCCT cohort even showed a numerically higher mean Agatston score. Thus, the superior assessability cannot readily be explained by a lower baseline calcium burden. Nevertheless, the study was not designed to isolate the individual contributions of detector technology, acquisition parameters, and reconstruction algorithms.

Combining improved anatomical imaging with functional assessment may further enhance the clinical utility of PCCT. In the CT2TAVI study, CT-FFR helped avoid downstream invasive testing in 60.5% of patients with obstructive proximal disease on CT angiography or at least one uninterpretable proximal segment [53]. Studies investigating PCCT-derived CT-FFR have reported promising results, including strong correlations with EID-CT-based measurements and high inter-reader agreement [54], [55], [56], [57]. However, these studies involved relatively small cohorts, and further validation is required. The combination of PCCT and CT-FFR may ultimately help identify patients who require ICA, but its clinical benefit in the TAVI population remains to be established.

The radiation-dose findings provide an additional potential advantage of PCCT. CT dose index, dose-length product, and estimated effective radiation dose were significantly lower with PCCT than with DSCT in our cohort. However, radiation dose was not the primary endpoint, and differences in acquisition and reconstruction protocols may have contributed to the observed results. These findings should therefore be interpreted as supportive rather than definitive evidence of a dose-specific advantage. Further protocol-standardized studies are needed to clarify the independent contribution of PCCT technology to dose reduction.

Several technical developments may further improve coronary assessment. Low-energy virtual monoenergetic imaging can improve vascular contrast and image quality [58], [59], while alternative contrast agents suitable for K-edge imaging may facilitate spectral separation of

tissue components and contrast materials [60], [61].

Several limitations of this study should be acknowledged. First, the study population was limited in size, and larger multicenter cohorts are needed to confirm the generalizability of the findings. Second, the examinations were acquired for TAVI planning rather than dedicated coronary assessment, limiting direct comparability with optimized coronary CT angiography. Third, ICA was not systematically performed as a reference standard; consequently, diagnostic accuracy and the safety of a CT-based strategy for deferring ICA could not be established. Fourth, the comparison reflects the overall imaging protocols used in the respective cohorts, and the independent effects of detector technology, acquisition parameters, and reconstruction methods cannot be fully separated. Finally, subsequent patient management and clinical outcomes were not systematically assessed. Whether improved assessability translates into fewer invasive examinations or better patient outcomes therefore remains unknown.

In conclusion, we are of the opinion that PCCT substantially improves coronary assessability and subjective image quality in non-coronary-dedicated TAVI planning CT compared with DSCT, despite lower quantitative SNR and CNR and without routine coronary premedication. Its principal advantage was the higher proportion of examinations that could be assessed diagnostically and assigned a CAD-RADS classification. These findings support the potential of PCCT to facilitate more comprehensive preprocedural coronary assessment. Prospective multicentre studies using standardized imaging protocols, an appropriate clinical reference standard, and systematic follow-up are required to determine whether improved assessability translates into higher diagnostic accuracy, fewer unnecessary invasive examinations, and meaningful improvements in patient management.

## CRediT authorship contribution statement

**Ines Mecking:** Writing – original draft, Visualization, Validation, Supervision, Software, Resources, Project administration, Methodology, Investigation, Funding acquisition, Formal analysis, Data curation, Conceptualization. **Marc von der Stück:** Writing – review & editing, Visualization, Validation, Supervision, Project administration, Methodology, Investigation, Formal analysis, Data curation, Conceptualization. **Eric Corban:** Visualization, Validation, Resources, Project administration, Methodology, Investigation, Formal analysis, Data curation, Conceptualization. **Michael Frick:** Validation, Supervision, Methodology, Investigation, Formal analysis, Conceptualization. **Jörg Schröder:** Resources, Project administration, Formal analysis, Data curation, Conceptualization. **Roman Iwa:** Resources, Project administration, Methodology, Formal analysis, Conceptualization. **Christiane K. Kuhl:** Validation, Resources, Project administration, Methodology, Investigation, Formal analysis, Data curation, Conceptualization. **Timm Dirrichs:** Writing – review & editing, Visualization, Validation, Supervision, Software, Resources, Project administration, Methodology, Investigation, Formal analysis, Data curation, Conceptualization.

## Ethics declaration

Written informed consent to take part in the study and to publish the article has been obtained from all participants or their legal representatives. The privacy rights of participants have been observed.

This study was performed in compliance with relevant laws, regulatory frameworks and guidelines where the research took place. This study was approved by the Ethics Committee University Hospital Aachen. (Approval No. EK 205–17)

## Ethical statement

Institutional Review Board approval was obtained from the ethical committee of the Medical Faculty of RWTH Aachen University (EK 286/19) on August 22nd, 2019, and was conducted in accordance with the Declaration of Helsinki. All participants provided written informed consent.

## Declaration of Competing Interest

The authors declare that they have no known competing financial interests or personal relationships that could have appeared to influence the work reported in this paper.

## References

[1] Yadgir (2020 May 26). Global Burden of Disease Study 2017 Nonrheumatic Valve Disease Collaborators. Global, Regional, and National Burden of Calcific Aortic Valve and Degenerative Mitral Valve Diseases, 1990-2017. Circulation.

[2] Yi B. (2021 May 11). Changing epidemiology of calcific aortic valve disease: 30-year trends of incidence, prevalence, and deaths across 204 countries and territories. Aging (Albany NY).

[3] Chen Q.F. (2024 Dec 17). Global, regional, and national burden of valvular heart disease, 1990–2021. J. Am. Heart Assoc..

[4] Boskovski M.T. (2021 Apr 30). Current therapeutic options in aortic stenosis. Circ. Res..

[5] Milin A.C. (2014 Sep 5). Insights into aortic sclerosis and its relationship with coronary artery disease. J. Am. Heart Assoc..

[6] Herrington W. (2016 Feb 19). Epidemiology of Atherosclerosis and the Potential to Reduce the Global Burden of Atherothrombotic Disease. Circ. Res..

[7] Vahanian A. (2022 Feb 12). ESC/EACTS Scientific Document Group. 2021 ESC/EACTS Guidelines for the management of valvular heart disease. Eur. Heart J..

[8] Otto C.M. (2021 Feb 2). 2020 ACC/AHA guideline for the management of patients with valvular heart disease: executive summary: a report of the american college of cardiology/american heart association joint committee on clinical practice guidelines. Circulation.

[9] Diller G.P., Gerwing M., Boroni Grazioli S., De-Torres-Alba F., Radke R.M., Vormbrock J., Baumgartner H., Kaleschke G., Orwat S. (2024 Jan 22). Utility of coronary computed tomography angiography in patients undergoing transcatheter aortic valve implantation: a meta-analysis and meta-regression based on published data from 7458 patients. J. Clin. Med..

[10] Becker L.M., Peper J., van Ginkel D.J., Overduin D.C., van Es H.W., Rensing B.J.M.W., Timmers L., Ten Berg J.M., Mohamed Hoesein F.A.A., Leiner T., Swaans M.J. (2025 Mar). Coronary CTA and CT-FFR in trans-catheter aortic valve implantation candidates: a systematic review and meta-analysis. Eur. Radiol..

[11] Reis J.F. (2022 Apr). Cardiac computed tomographic angiography after abnormal ischemia test as a gatekeeper to invasive coronary angiography. Int. J. Cardiovasc. Imaging.

[12] Dewey M. (2016 Oct 24). Evaluation of computed tomography in patients with atypical angina or chest pain clinically referred for invasive coronary angiography: randomised controlled trial. BMJ.

[13] Chang H.J. (2019 Jul). Selective referral using CCTA versus direct referral for individuals referred to invasive coronary angiography for suspected CAD. A Random Control. Open. -Label. Trial JACC Cardiovasc. Imaging.

[14] SCOT-HEART Investigators Newby D.E. (2018 Sep 6). Coronary CT Angiography and 5-Year Risk of Myocardial Infarction. N. Engl. J. Med..

[15] Cury R.C. (2022). CAD-RADS™ 2.0 – 2022 Coronary Artery Disease-Reporting and Data System: an expert consensus document of the society of cardiovascular computed tomography (SCCT), the American College of Cardiology (ACC), the American College of Radiology (ACR), and the North America Society of Cardiovascular Imaging (NASCI). J. Cardiovasc. Comput. Tomogr..

[16] Chiocchi M., Ricci F., Pasqualetto M., D'Errico F., Benelli L., Pugliese L., Cavallo A.U., Forcina M., Presicce M., De Stasio V., Di Donna C., Di Tosto F., Spiritigliozzi L., Floris R., Romeo F. (2020 Mar). Role of computed tomography in transcatheter aortic valve implantation and valve-in-valve implantation: complete review of preprocedural and postprocedural imaging. J. Cardiovasc. Med. (Hagerstown).

[17] Gatti M., Gallone G., Poggi V., Bruno F., Serafini A., Depaoli A., De Filippo O., Conrotto F., Darvizeh F., Faletti R., De Ferrari G.M., Fonio P., D'Ascenzo F. (2022 Aug). Diagnostic accuracy of coronary computed tomography angiography for the evaluation of obstructive coronary artery disease in patients referred for transcatheter aortic valve implantation: a systematic review and meta-analysis. Eur. Radiol..

[18] Renker M., Schoepf U.J., Kim W.K. (2023 Apr 3). Combined CT Coronary Artery Assessment and TAVI Planning. Diagn. (Basel).

[19] Gohmann R.F., Lauten P., Seitz P., Krieghoff C., Lücke C., Gottschling S., Mende M., Weiß S., Wilde J., Kiefer P., Noack T., Desch S., Holzhey D., Borger M.A., Thiele H., Abdel-Wahab M., Gutberlet M. (2020 May 27). Combined Coronary CT-Angiography and TAVI-Planning: A Contrast-Neutral Routine Approach for Ruling-out Significant Coronary Artery Disease. J. Clin. Med..

[20] Symons R., Reich D.S., Bagheri M., Cork T.E., Krauss B., Ulzheimer S., Kappler S., Bluemke D.A., Pourmorteza A. (2018 Mar). Photon-Counting Computed Tomography for Vascular Imaging of the Head and Neck: First In Vivo Human Results. Invest. Radiol..

[21] van der Bie J. (2024 Apr). Thin slice photon-counting CT coronary angiography compared to conventional CT: objective image quality and clinical radiation dose assessment. Med. Phys..

[22] Rønning M., Johansen E., Rusandu A. (2025 Jul 25). Photon-counting CT versus energy-integrating detectors for cardiac imaging: a systematic review of evidence from in vivo human studies on image quality and radiation dose. BMC Med. Imaging.

[23] van der Bie J., van der Laan T., van Straten M., Booij R., Bos D., Dijkshoorn M.L., Hirsch A., Oei E.H.G., Budde R.P.J. (2025 Sep). Photon-counting CT: an updated review of clinical results. Eur. J. Radiol..

[24] Schwartz F.R. (2023 Jan 1). Coronary artery calcium evaluation using new generation photon-counting computed tomography yields lower radiation dose compared with standard computed tomography. J. Thorac. Imaging.

[25] Brendel J.M., Walterspiel J., Hagen F., Kübler J., Paul J.F., Nikolaou K., Gawaz M., Greulich S., Krumm P., Winkelmann M. (2024 Jul). Coronary artery disease evaluation during transcatheter aortic valve replacement work-up using photon-counting CT and artificial intelligence. Diagn. Interv. Imaging.

[26] Sharma S.P., Verhemel S., Hirsch A., van der Bie J., Dijkshoorn M.L., Daemen J., van Mieghem N., Budde R.P.J. (2026 Mar). Diagnostic performance of high and ultra-high-resolution photon counting CT for detection of coronary artery disease in patients evaluated for transcatheter aortic valve implantation. Int. J. Cardiovasc. Imaging.

[27] Smolka S., Moshage M., Marwan M., Achenbach S. (2024). Visualization of the coronary arteries by photon counting CT in data sets acquired for TAVI planning: Analysis of image quality for assessing coronary artery disease. Clin. Res. Cardiol..

[28] Dirrichs T. (2024 Dec). Photon-Counting Versus Dual-Source CT for Transcatheter Aortic Valve Implantation Planning. Acad. Radiol..

[29] Blanke P. (2019 Jan). Computed Tomography Imaging in the Context of Transcatheter Aortic Valve Implantation (TAVI)/Transcatheter Aortic Valve Replacement (TAVR). Expert. Consens. Doc. Soc. Cardiovasc. Comput. Tomogr. JACC Cardiovasc. Imaging.

[30] Callister T.Q., Cooil B., Raya S.P. (1998). Coronary artery disease: improved reproducibility of calcium scoring with an electron-beam CT volumetric method. Radiology.

[31] Agatston A.S., Janowitz W.R., Hildner F.J. (1990). Quantification of coronary artery calcium using ultrafast computed tomography. J. Am. Coll. Cardiol..

[32] Jessen, K.A., et al; Directorate-general for research and innovation, European Commission. European Guidelines on quality criteria for computed tomography Office for Official Publications of the European Communities, Luxembourg City, Luxembourg, 2000.

[33] Shnayien S. (2020). Radiation dose reduction in preprocedural CT imaging for TAVI/TAVR using a novel 3-phase protocol: a single institution's experience. Rofo.

[34] Kędzierski B. (2023). Radiation doses in cardiovascular computed tomography. Life.

[35] Manoukian S.V. (2009). Predictors and Impact of Bleeding Complications in Percutaneous Coronary Intervention, Acute Coronary Syndromes, and ST-Segment Elevation Myocardial Infarction. Am. J. Cardiol..

[36] Applegate R. (2009). Prognostic implications of vascular complications following PCI. Catheter. Cardiovasc. Interv..

[37] Applegate R.J. (2008). Trends in Vascular Complications After Diagnostic Cardiac Catheterization and Percutaneous Coronary Intervention Via the Femoral Artery, 1998–2007. JACC Cardiovasc. Interv..

[38] Giannini F. (2018). A practical approach to the management of complications during percutaneous coronary intervention. JACC Cardiovasc. Interv..

[39] Morgan-Hughes G. (2021). Downstream Testing After CT Coronary Angiography: Time for a Rethink?. Open. Heart.

[40] Bolognese L., Reccia M.R. (2022). Computed Tomography to Replace Invasive Coronary Angiography? The DISCHARGE Trial. Eur. Heart J. Suppl..

[41] Douglas P.S. (2016). PLATFORM Investigators. 1-Year Outcomes of FFRCT-Guided Care in Patients With Suspected Coronary Disease: The PLATFORM Study. J. Am. Coll. Cardiol..

[42] Sakai K. (2025). Diagnostic Performance and Clinical Impact of Photon-Counting Detector Computed Tomography in Coronary Artery Disease. J. Am. Coll. Cardiol..

[43] Haag N.P. (2024). Coronary Artery Calcium Scoring Using Virtual Versus True Non-Contrast Images from Photon-Counting Coronary CT Angiography. Radiology.

[44] Vecsey-Nagy M. (2023). Calcium scoring on coronary computed tomography angiography with photon-counting detector technology: predictors of performance. J. Cardiovasc. Comput. Tomogr..

[45] Koons E.K. (2024). Coronary artery stenosis quantification in patients with dense calcifications using ultra-high-resolution photon-counting-detector computed tomography. J. Cardiovasc. Comput. Tomogr..

[46] Halfmann M.C. (2024). Ultrahigh-Spatial-Resolution Photon-Counting Detector CT Angiography of Coronary Artery Disease for Stenosis Assessment. Radiology.

[47] McCollough C.H. (2023). Comparison of Photon-Counting Detector and Energy-Integrating Detector CT for Visual Estimation of Coronary Percent Luminal Stenosis. Radiology.

[48] Wolf E.V. (2023). Intra-Individual Comparison of Coronary Artery Stenosis Measurements Between Energy-Integrating Detector CT and Photon-Counting Detector CT. Imaging.

[49] Mancini G.B.J. (2021). CT Angiography Followed by Invasive Angiography in Patients With Moderate or Severe Ischemia—Insights From the ISCHEMIA Trial. JACC Cardiovasc. Imaging.

[50] Mergen V. (2022). Ultra-High-Resolution Coronary CT Angiography with Photon-Counting Detector CT: Feasibility and Image Characterization. Invest. Radiol..

[51] Hagar M.T. (2023). Accuracy of Ultrahigh-Resolution Photon-Counting CT for Detecting Coronary Artery Disease in a High-Risk Population. Radiology.

[52] Eberhard M. (2024). Coronary Stenosis Quantification With Ultra-High-Resolution Photon-Counting Detector CT Angiography: comparison With 3D Quantitative Coronary Angiography. JACC Cardiovasc. Imaging.

[53] Hussain K. (2025). Real-World Application of CCTA with CT-FFR for Coronary Assessment Pre-TAVI: The CT2TAVI Study. Int. J. Cardiovasc. Imaging.

[54] Tremamunno G. (2025). Influence of Virtual Monoenergetic Reconstructions on Coronary CT Angiography-Based Fractional Flow Reserve with Photon-Counting Detector CT: intra-individual comparison with energy-integrating detector CT. Insights into Imaging.

[55] Vecsey-Nagy M. (2024). Coronary CT Angiography-Based FFR with Ultrahigh-Resolution Photon-Counting Detector CT: Intra-Individual Comparison to Energy-Integrating Detector CT. Eur. J. Radiol..

[56] Zsarnoczay E. (2024). Intra-Individual Comparison of Coronary CT Angiography-Based FFR Between Energy-Integrating and Photon-Counting Detector CT Systems. Int. J. Cardiol..

[57] Sartoretti T. (2023). Photon-Counting Detector Coronary CT Angiography: Impact of Virtual Monoenergetic Imaging and Iterative Reconstruction on Image Quality. Br. J. Radiol..

[58] Jost G. (2023). New Contrast Media for K-Edge Imaging With Photon-Counting Detector CT. Invest. Radiol..

[59] Booz C. (2024). Carotid Artery Assessment in Dual-Source Photon-Counting CT: Impact of Low-Energy Virtual Monoenergetic Imaging on Image Quality, Vascular Contrast and Diagnostic Assessability. Radiol. Med..

[60] Zsarnoczay E. (2024). Intra-Individual Comparison of Coronary CT Angiography-Based FFR Between Energy-Integrating and Photon-Counting Detector CT Systems. Int. J. Cardiol..

[61] Jost G. (2023). New Contrast Media for K-Edge Imaging With Photon-Counting Detector CT. Invest. Radiol..

